# 

**Published:** 1897-07

**Authors:** 


### BOOKS RECEIVED.

New Volume of Hare's System of Practical Therapeutics. A System of Practical Therapeutics. By Eminent Authors. Edited by Hobart Amory Hare, M. D., Professor of Therapeutics and Materia Medica in the Jefferson Medical College of Philadelphia. Volume IV. Octavo, 1,065 pages, with illustrations. Regular price, cloth, $6 ; leather, $7 ; half Russia, $8. Price of Volume IV. to subscribers to the System, cloth, $5 ; leather, $6 ; half Russia, $7. Price of the System complete in four volumes of about 4,500 pages, with about 550 engravings, cloth, $20 ; leather, $24 ; half Russia, $28. Philadelphia and New York : Lea Brothers & Co., Publishers. 1897.

Medical and Surgical Report of the Presbyterian Hospital in the City of New York. Volume II. January, 1897. Edited by Andrew J. McCosh, M. D., and Walter B. James, M. D. New York. 1897.

Reports of Inspections of National, State and Local Quarantine Stations. From annual report Marine Hospital Service. Washington : Government Printing Office. 1897.

The Disorders of Digestion in Infancy and Childhood. By W. Soltau Fenwick, M. D., B. S., London, Member of the Royal College of Physicians, etc. Illustrated. Philadelphia : J. B. Lippincott Company. 1897.

The Medical Directory of the City of New York. Published under the auspices of the Medical Society of the County of New York. Daniel Lewis, M. D., Editor. Price, $1. New York : Press of Le Huray & Co., 14 Reade street. 1897.

Hysteria and Certain Allied Conditions. Their Nature and Treatment, with Special Reference to the Application of the Rest Cure, Massage, Electrotherapy, Hypnotism, etc. By George J. Preston, M. D., Professor of Diseases of the Nervous System, College of Physicians and Surgeons, Baltimore ; Visiting Physician to the City Hospital, etc., etc. Small 8vo, pp. 298. Illustrated. Price, $2. Philadelphia : P. Blakiston, Son & Co., 1012 Walnut street. 1897.

---

### Literary Notes.

### INDEX TO VOLUME XXXVI.

PAGE.

ABDOMEN, Contusions of . 302
Abdominal Surgeons' Operations, Cleanliness in . . . . . . 483
Section vs. the Vaginal Route in Periuterine Septic Diseases . . . 743
Acetonuria . . . . . . . . . . . . 36
Acromegaly, Pathological Study of . 605
Advertising Tramp, Conviction of . 159
Albumin, Simple Test for . . . . 350
Albuminuria . . . . . . . . . . . 596
in Pregnant Women . 353
American Public Health Association, History of the Preparation for the Buffalo Meeting . . . 105
Public Health Association, Effects of . . . 226
Medical Association, Notes and Echoes of the Jubilee Meeting . 928
Anchylostamum Duodenale . . . 573
Anesthesia in Surgery, History of . 241
Semicentennial Anniversary at Massachusetts General Hospital . . . . . . . . . . 315
Gilbert Abbott and . . 316
Anesthetic, Local . . . . . . . . 852
Angell, E. B., Sciatica a Neuritis, Rarely a Neuralgia . . . . . . 410
Antitoxin in Private Practice . . . 119
Note on the Use of . . . 125
Antivivisectionists, Campaign of . 536
Appendicitis, Nonoperative Treatment in . . . . . . 684
Fulminating . . . . 684
Arecolin, Physiological Action of . 365
Argonin in Gonorrhea . . . . 351
Astasia-Abasia, Cases of . . . . 856
Asthenopia, Rational Treatment of . 580
Astigmatism, Operative Treatment of . . . . . . . . . . . . . . . 30
Astrologists and Fortune Tellers Taxed at Cleveland . . . . . . 160
Ataxia, Two Cases of Friedrich's . 568
Atropine in Tinnitus Aurium of Quinine . . . . . . . . . . . 850

PAGE.

BACK to Nature . . . . . . . . 323
Bartlett, Frederick W., In Memory of . . . . . . . . . . . 679
Barton, Clara . . . . . . . . . . 377
Basedow's Disease Surgically Treated . . . . . . . . . . . . . 604
Benzosol, Clinical Applications of . 308
Bicycling and Breathing . . . . . 339
a Danger of . . . 601
Bills, Mischievous at Albany . . . 615
Blood, Oxidising Power of . . . . 36
Books Received . . . 76, 156, 237, 321, 397, 479, 559, 636, 721, 796, 882, 945
Brain Syphilis, Anatomical Consideration of . . . . . . . . . . . 643
Breast Replaced by Lipoma . . . 773
Burns, Treatment of . . . . . . 303

CALCULUS, Diagnosis of by the New Photography . . . . 348
Camphoric Acid in Night Sweats . 853
Carpenter, Thomas B, Casts of Uriniferous Tubules . . . . . . 1
Castration for Prostatic Hypertrophy . . . . . . . . . . . . . 350
Cataract Extraction . . . . . . . 360
Cervix, Lacerations of . . . . . . 768
Cesarean Section . . . . . . . . 773
Chancre, Extra-genital . . . . . . 671
Chautauqua County Notes . . 385, 877
Cheesman, Wm. S., Back to Nature . 323
Children, Employment of in Offices. 375
Christian Science Fad . . . . . 61
and Schlatterism . 376
Another Set-Back for . . . . 460
A Knock-out of . 613
Ciliary Body, Steel Located in by Röntgen Rays . . . . . . . . . 766
Clark, Horace, Reflex Effects of Intranasal Disease in the Pharynx and Mouth . . 415
Ailments of Voice Users . 920
Clarke, Gardner Carpenter, Memorial of . . . . . . . . . . . . 873
Clemesha, J C., Note on Eucaine . 182
Acute Pleural Effusion in Children . . . . . 331

PAGE.
Clemesha, Eucaine "B" etc . . . 845
Cocaine, Ordinance against . . 850
Cod-liver Oil, Substitute for . . . 601
Consumption, Control of . . . . . 535
Deaths from . . . 781
Continued Fever, not Typhoid . . 450
Convalescent Dinner Society . . . 130

**Correspondence:**

Comments on Dr. Rider's Paper, D. B. St. John Roosa. 692
Double Uterus and Vagina, Frank A. Jones . . . . . 530
Letter from William Wood & Co . . . . . . . . . . . 696, 775
Monopolistic Control of Medical Literature, George M. Gould . . . . . . . . . 694, 774
Mr. Rafter and Lake Erie Water Supply, W. G. Bissell. 307
Tuberculosis Conveyed by Milk, J. M O'Neill . . 607
Crockett, M. A., Case of Uterine Myoma . . . . . . . 511
Etiology and Pathology of Uterine Displacements . 667
Cumston, Charles Greene, Pathology of Congenital Hydrocele . . 907

DAGENAIS, A., Note on the Use of Antitoxin . . 125
Alphonse, In Memory of . . . 675
Damages, Prosecution for in Abdominal Surgery . . . . . . . 160
Death-rate at Buffalo . . . . . . . 313
Dental Department, U. of B., Dedication of New Building . . . . 373
Diabetes, Oxygen in . . . . . . . 529
Forms of . . . . . . . 606
Diarrhea, Simple . . . . . . . . . 126
Summer in Infants . . . 600
Diehl, Alfred E , Extra-genital Chancre . . . . . . . . . . . . 671
Diphtheria of the Naso-pharynx . 438
Antitoxin Treatment of in Buffalo . . . . . . 723
Diphtheritic Paralysis . . . . . . 219
Dispensary, Abuse of the . . . . 925
and Hospital Abuse at Washington . . . . 456
Dowd, J. Henry, Inflammatory Disease of the Prostate, etc . . . 491
Drug Company, New at Buffalo . 160
Dunham, Sidney A., Involuntary Intoxication from a Medico-legal Standpoint . . . . . . . . . . 99

PAGE.
EAR, Objective Noise in . . . 181
Earache, Its Significance and Management . . . . . . . . . 727
Echinococcus Cysts, Case of Multiple . . . . . . . . . . . . 25
Ehrlich's Diazo Reaction . . . . . 37
Elsner, S L , Points in After-treatment of Surgical Cases . . . 836
Enteroptosis, Some Aspects of . . 895
Enuresis . . . . . . . . . . . 40
Ephedrene as a Mydriatic . . . . 54
Epilepsy, Operative Treatment of . 304
Epileptic Patients, Colonies for . . 608
Epiphysitis, Acute Nontubercular
Ether Anesthesia, History and Semicentennial Anniversary . . . . . . . . . . . 266
Administration of to Infants. 436
Ethics in Our Colleges . . . . . . 538
Eucaine . . . . . . . . . . . . . 764
Note on . . . . . . . . . 182
in Ophthalmic Practice . 363
Local Anesthesia by . . . 595
"B." . . . . . . . . . . 845
in Infants . . . . . . . . . . . 161
Examinations, Entrance . . . . . 445
Examining Board, Report of New York State . . 588
Pennsylvania State . . . . 593
Experts, Medical and Expert Testimony . . . . . . . . . . . . . 403
Eye Specimens, Preparation of . . 51

FECAL Fistula, Treatment of . 530
Feces, Clinical Importance of an Examination of . . . . . 89
Ferripyrine . . . . . . . . . 768
Fever, an Epidemic of Glandular . 602
Fibrin, Digestion of . . . . . . . 36
Flies, How to Drive Away . . . . 158
Formalin in Genito-urinary Diseases . . . . . . . . . . . 349
Formic Aldehyde in Ophthalmic Practice . . . . . . . . . . . 55
Formulæ . . . . . . . . . . . . . 852
Frick Library, Opening of at Baltimore . . . . . . . . . . . 457
Frye, Maud J., Woman's Place in Medicine . . . . . . . . . . . 868

GALL-STONE, Cases of . . 855 . .
Gastrostomy for Stricture of the Esophagus . . . . . . . . 81
Goitre, Thyroidectomy for Relief of Exophthalmic . . . . . . . 606
Gonococcus Pyemia . . . . . . . 597

PAGE.
Gonorrhea in Women . . . . . . 356
Argonin in . . . . . . 691
Gross Statue, Unveiling of . . . . 534

HAUENSTEIN, John, Résumé of Fifty Years' Obstetric Practice . . . . . . . . . . . 810
Heartstrain . . . . . . . . . . . 306
Hemorrhages, Degenerations Following Focal . . . . . . . . . 604
Herpes Facialis . . . . . . . . . 651
Hiccough, Paroxysms of . . . . . 849
Himmelsbach, G. A, Pregnancy Following Imperfect Coitus . 347
Hinkel, F. Whitehill, Earache, Its Significance and Early Management . . . . . . . . . . 727
Holocain, A New Local Anesthetic. 763
Hubbell, A. A., Measures to Prevent Ophthalmia Neonatorum . . . . . . . . 426
Should Opticians Practise Medicine? . . . . . . 741
Hurd, Arthur W., Prevention and Early Treatment of Mental Disease . . . . . . . . . . . . . 168
Hydramnios and its Complications. 767
Hydrocele, Pathology of Congenital . . . . . . . . . . . . . . 907
Hysterectomy in Inflammatory Diseases . . . . . . . . . . . . . 767
Hysteria . . . . . . . . . . . . 504

IDAHO, Medical Law of . . . . 862
Ileus, Dynamic . . . . . . . 293
Illinois State Board of Health, Resolutions of . . . . . . . . . 859

**Illustrations:**

Alphonse Dagenais, M. D. . . 709
American Public Health Association (Potter), Hotel Niagara . . . . . . . . . . 107
Alfred A. Woodhull . . . 115
Buffalo Library . . . . . 111
Eduardo Licéaga . . . . 114
Ellicott Square . . . . . 109
Henry D. Holton . . . . 118
Henry Sewall . . . . . . 116
Irving A. Watson . . . . 117
View on Delaware Avenue 113
Anesthesia in Surgery (Park), Wm. T. G. Morton, Facing . 241
Bath House, Hot Springs, Va. 312
Chairman of Committees American Public Health Association, Facing . . . . . . . . 222
Clara Barton . . . . . . . . . 377
Ernest Wende, Health Commissioner . . . . . . . . . . 454
Ernest Wende, Portrait of, Facing . . . . . . . . 222
First Demonstration of Etherisation . . . . . . . . . . . 253
Friedreich's Ataxia (Stephenson) 4 figures . . . . . . . . 571
Gardner Carpenter Clarke, M. D. . . . . . . . . . . . . 712
German Deaconess's Home and Hospital . . . . . . . . . 462
Hon. James O. Putnam . . . 263
Modern Gastrostomy (McMurtry), Figures 1 and 2 . . . . 83
Figure 3 . . . . . . . . 84
Figure 4 . . . . . . . . . 85
Figures 5, 6 . . . . . . . . 86
Figure 7 . . . . . . . . 88
Mycosis Fungoides (G. W. Wende) . . . . . . . . 834
Old Amphitheatre, Massachusetts General Hospital . . . 257
Right Kidney with Cyst (Keyes-Busch), Figure 1 . . 27
Scolex with Hooplets and Brood Capsules, Figure 2 . 27
Walter D. Greene, Deputy Health Commissioner . . . 454
Impetigo, Contagious . . . . . . . 602
Infants, Drooling of . . . . . . . . 194
Tubercolosis in . . . . . 925
Inguino-scrotal Region, Formations in . . . . . . . . . . . . . . . 41
Insanity in Women, Causes of . . 298
Intoxication, Involuntary, from a Medico-legal Standpoint . . . . 99
Intraäbdominal Disease, Deceptive Similarity of Symptoms of . . . 283
Intranasal Disease, Reflex Effects of . . . . . . . . . . . . . 415
Iris, Coloboma of . . . . . . . . . 280
Iron as Food . . . . . . . . 33
Formation of Blood from . . 34

**Items:**

A L. Hummel Advertising Agency . . . . . . . . . . . 802
Brains Wanted . . . . . . . 240
Charles Roome Parmele Co. . 642
Climate and Health . . . . 240
Conferences Local Boards of Health . . . . . . . . . . . 133
Course in Bacteriology . . . . 641
Dispensary Abuse . . . . 946
Emmet Library, Sale of . . . 134
Gross Statue, Unveiling of . . 801

PAGE.

Herbert U. Williams's Spring Course . . . . . . . . . . . 642
Hermann Munk, Du Bois Reymond's Successor . . . . . 642
Medical Press Bureau . . 802
Mellin's Food in Infantile Diarrhea . . . . . . . . . . 642
Monument to Pasteur . . . . . 80
New York Lying-in-Hospital, Gift of J. Pierpont Morgan to. . . . . . . . . . . . . 641
New York Physicians' Mutual Aid Association Report . . . 802
Pajot, Witticisms of . . . . . 134
Physicians in Paris and New York . . . . . . . . . . 240
Skiagraph, Legal Status of . . 240
Special Tour to Europe . . . 802
Trustees' Report American Medical Association . . . 131

JACOBSON, Nathan, History and Semicentennial Anniversary of Anesthesia . . . . . . 266
Johnston, George Ben., Address by . 933
Joint Affections, Treatment of Chronic . . . . . . . . . . . . 305

KERATITIS, Suppurative . . . 55
Keyes-Busch, Multiple Echinococcus Cysts . . . . . . . . . 25
Kidney, Operation for Sacculated . 123
Movable . . . . . . . . 287
Disease, Lactate of Strontium in . . . . . 597
Koch's Mission to South Africa . . 613
Krauss, William C., Relation of Noises to Public Health. 184
Anatomical Consideration of Brain Syphilis . . . 643

LAMP, Portable Spirit . . . . 9

**Leading Articles:**

American Public Health Association . . . . . . . . . 57, 222
American Association of Obstetricians and Gynecologists . . . . . . . . . . . . 311
American Medical Association, Jubilee Meeting . . . . . 926
Anesthesia, Semicentennial Anniversary of . . . . . . . . 309

PAGE.

Annual Commencement, Buffalo University Medical College . . . . . . . . . . 776
Annual Commencement Niagara University Medical College . . . . . . . . . . . . 864
Buffalo Sanatory Club . . . . 609
Dangers of Bicycling . . . . 867
Health Department, Its New Appointees . . . . . . . . 453
Medical Society State of New York . . . . . . . . . . . 610
National Confederation of State Medical Examining and Licensing Boards . . . 931
Opening of the Colleges 127
Prevention of Disease by Municipal Employment . . . . 370
Relation of Drinking Water to Health . . . . . . . . . . 531
The Year . . . . . . . . . . 451
The Plague . . . . . . . . . 702
Legislation at Albany . . . . . . 671
Library, Buffalo Free Public . 374, 459

**Literary Notes:**

Addendum to Lawson Tait's Paper . . . . . . . . . . . . 945
Albany Medical Annals . . . 722
American Journal of Insanity . 946
American Medical Journalist . 562
Anales d'Oculistique . . . . 480
Anesthesia, a Photogravure of. 399
Anomalies and Curiosities of Medicine . . . . . . 482
Antikamnia's Christmas Souvenir . . . . . . . . . . . . 481
Archives of Clinical Skiagraphy . . . . . . . . . . . 80
Boletin da Sociedade de Medicina e Cirurgia de S Paulo 799
Buffalo General Hospital Annual Report . . . . . . . . . 801
Canadian Journal of Medicine and Surgery . . . . . . . . 562
Columbia Calendar . . . . . 482
Columbus Medical Journal . . 799
Consolidation of Medical Journals . . . . . . . . . . . 562
Electrical Review . . . . . 79
Fort Wayne Medical Journal-Magazine . . . . . . . . . . 798
Gray's Anatomy . . . . . . . 157
Greater Buffalo . . . . . . 946
Illinois State Reformatory, Third Biennial Report . . . 800
Intercollegiate Medical Journal 799
International Medical Annual . 638

PAGE.
Jackson Sanatorium . . . . . 800
Journal of Nervous and Mental Diseases . . . . . . . . 399
Journal of Cutaneous and Genito-urinary Disease . . . . . 639
Laryngoscope . . . . . . . . 79
Mathews's Medical Quarterly . 322
Mellier Drug Company's Brochure . . . . . . . . . . . . 481
Medical History of Tennessee . 637
Medical Compend . . . . . . 562
Medical Mirror . . . . . . . 481
Medical Press Bureau . . . . 157
Medical Record of Mississippi . . . . . . . . . . . 799
Military Cycling in the Rocky Mountains . . . . . . . 798
New York Medical Journal . . 80
Ophthalmic Record . . . . . 480
Physician's Blue Book . . . . 800
Physician's Vest Pocket Formula Book . . . . . . . 640
Report of New York Pysicians' Mutual Aid Association . . 79
Resection of Blood Vessels, End-to-End suture (Murphy) . . . . . . . . . . . 800
Revista de Ciencias Medicas de Barcelona . . . . . . . 800
Scientific American . . . . 78
Sheppard Asylum, Fifth Annual Report . . . . . . . . . 798
State Requirements for the Practice of Medicine . . . . 638
Students' Hand-Book . . . . 79
Wilkinson's Medical Magazine . 80
Take Your Home Medical Journal . . . . . . . . . 398
Therapeutic Progress . . . . 480
Treat, E. B. & Co . . . . 946
Visiting Graduate Nurses . . . 482
Western Medical Review . . 78
Warner's Pocket Medical Dictionary . . . 639
Year Book of the New York State Reformatory . . . . . 639
The Laryngoscope . . . . . . 640
Ten-cent Journalism . . . . . 640
Treatment . . . . . . . . . 799
Year's Work in Operative Gynecology (Humiston) . . 800

Lithia Tablets . . . . . . . . . 56
Litholapaxy, Points in Technique of . . . . . . . . . . . . . 211
Love, Dr. I. N., Resignation of . . 129
Loveland, B. C., Hysteria . . . . 504
Lumbar Puncture for Relief of Intracranial Pressure . . . . . 215
Lupus Erythematosus . . . . . . 853

PAGE.
MANN, Matthew D, Fibroid Operation by . . . . . . . 923
Maryland Examining Board . . . 447
Marriage Contract, Should it be Limited by Law? . . . 733
and Social Purity . . . . 771
McMurtry, Lewis S, Modern Gastrostomy for Stricture of the Esophagus, with Case . . . . . 81
Medical Colleges, Standing of . . . 44
Examinations, Division of . 46
Schools, Inspector of . 444
Science, Practical Aspects of Modern . . 656
Practice Act, Struggles and Trials of . . . . . 703
Legislation in the United States . . . . . . . . . 857
Student Examinations in Illinois . . . . . . . . . 862

**Medical College, Hospital and Dispensary Notes:**

Albany Medical College . . . 384
Bellevue Hospital Medical College . . . . . . . . . . 545
Buffalo Fresh Air Mission Hospital . . . . . . 60
Buffalo State Hospital . . . . 69
Buffalo City Hospital for Women . . . . . . . . 70
College of Physicians and Surgeons, Chicago . . . 788
German Deaconess's Home and Hospital . . . . . . . . 461
German Dispensary . . . . 463
Kentucky School of Medicine . 144
Marion Sims College of Medicine . . . . . . . . . . . 144
New York University—Bellevue Hospital Medical College . . . . . . . . . 788, 875
New York Post-Graduate Medical School and Hospital. 69, 621
Niagara University Medical College . . . . . . . . . 788
Pennsylvania State Consumption Sanitarium . . . . . 546
Riverside Hospital, Opening of New . . . . . . . . 225, 877
Melano-sarcoma of the Female Urethra . . . . . . . . . . 344
Melena, Theory to Account for . . 438
Mental Disease, Prevention and Early Treatment of . . . . . . 168
Mexican Delegates at Buffalo Meeting of American Public Health Association . . . . . . . . . . 226

PAGE.
Microscope, The Physician and His. 195
Midwife, Conviction of in Maryland . . . . . . . . . . . . . 458
Milk, Pasteurisation of . . . . . . 121

**Miscellany:**

Antitoxin Collective Investigation . . . . . . . . . . . 399
Arlington Chemical Co. Prize Contest . . . . . . . . . 402
Book of Delinquent Debtors . 402
Peroxide of Hydrogen, Marchand . . . . . . . . . . . 402
Rush Monument . . . . . . . 400
Caroid . . . . . . . . . . 401
Subscribers in Arrears . . . . 402

Missouri, Requirements in . . . . 443
Möhlau, F. H., Anchylostamum Duodenale, with Report of Cases. 573
Morton, Wm. T. G., in Buffalo . . 264
Murphy Button . . . . . . . . . 782
Mycosis Fungoides, with Case . . 832

NATURE Study in Schools . . 780
Nephrorrhaphy, Limits of . 288
Nephritis, Pilocarpin in Treatment of . . . . . . . . . 691
New York State Medical Association, Change of Front of . . . . . . . 704
Physicians' Mutual Aid Association . . . . . 708
Nitroglycerin for Gall-stone . . . 221
Noises, Relation of to Public Health . . . . . . . 184
Suppression of Unnecessary . . . . . . . . 533
Nursing Bottle Ordinance in Buffalo . . . . . . . . . . . . 614

**Obituary:**

Barross, F. R. . . . . . . . . . . 136
Bartlett, F. W. . . . . . . 710
Brennan, Daniel H. . . . 137
Burchard, Thomas Herring . . 380
Churchill, Alonzo . . . . . 542
Clarke, Gardner C. . . . . . 711
Cochran, Jerome . . . . . . 135
Dagenais, Alphonse . . . . 708
Erichsen, John Eric . . . . . 381
Grant, James A. S. . . . . . 135
Green, Traill . . . . . . . . 873
Hays, James Mackintosh . . . 872
Hunn, Thomas . . . . . . . 65
Lewis, John C. . . . . . . . 618
Lusk, William Thompson . . 935

PAGE.
McLean, Leroy . . . . . . . 783
Millard, Perry H. . . . . . . 617
Murdoch, James Bissett . . . 381
Pancoast, William H. . . . 541
Potter, Samuel . . . . . . . 938
Richardson, Benjamin Ward . 380
Samo, James B. . . . . . 710
Smith, James Greig . . . . . 937
J. Lewis . . . . . . . 936
Walker, Francis Amasa . . . 538
Wells, Sir Spencer . . . . . . 616

Obstetric Practice, Résumé of Fifty Years' . . . . . . . . . . . . 810
Ohio Medical Practice Act . . . . 442
Ointments, Decomposition of in Light . . . . . . . . . . . 53
Ophthalmia Neonatorum, Prevention of . . . . . . . . 426
Opticians, Should They Practise Medicine? . . . . . . . . . . . 741
Orchitis, Bismuth Paste in . . . 701
Ovariotomist, Was Robert Houston the First? . . . . . . . . 803
Ovary, Mixed Tumors of . . . . . 287

PALMER, John O., Practical Aspects of Modern Medical Science . . . . . . . . . . . . 656
Pan-American Medical Congress . 233
Parasites in the Vermiform Appendix . . . . . . . . . . . . 176
Park, Roswell, History and Introduction of Anesthesia in Surgery . . . . . . . . . 241
Pasteur Monument . . . . . . . 535
Pediatrics, Past, Present and Prospective . . . . . . . . . . . . 435
Pelvic Disease, Cause of . . . . . 282
Inflammation, Diffused . . 355
Inflammation, Potent Causes of . . . . . . . . . . . 284
Peptone, Intestinal Absorption of . 37
Perineal Laceration . . . . . . 356
Peritonitis, Laparatomy and Drainage in Purulent . . . . . . . 509
Periuterine Septic Diseases, Abdominal Section vs. Vaginal Route . . . . . . . . . . 292, 743

**Personal:**

Blackwell, Elizabeth; Abbott, A. C.; Smith, Chauncey P.; McCutcheon, Guy L.; Dignen, W. E. . . . . . . . 545
Bryant, Percy . . . . . . . . 562
Coleman, N. R. . . . . . . . 938

PAGE.

Didama, H. D.; Peterson, Frederick C.; Hamilton, John B.. . . . . . . . . . . 939
Da Costa, J C.; Clark, Edw.; Cott, Geo. F.; Clark, Horace; Thornbury, F. J. . . . 713
Frost, Henry P. . . . . . . . 616
Hamilton, John B.; Pitkin, Jno. T.; Goldberg, J.; Vandenbergh, Frank P.; Bissell, Wm G. . . . . . . . . . . . 317
Hooper, P. O.; Colvin, Darwin; Fite, C. C. . . . . 460
Krauss, William C.; Wilson, A B. . . . . . . . . . . . . 714
Lewis, Daniel; Ogden, Charles L.; Schade, L; Keiser, Max . 461
Lewis, Daniel; Weigel, L. A.; Starr, Elmer; Gibson, Jas. A.; Kane, J J. . . . . 872
Leonhardt, H. C; Ross, Jas. F. W.; Caul, Martha F.; Patterson, C. J.; Hare, H. A; Bates, N. L . . . . . . 783
Lister, Sir Joseph . . . . . . 544
Letchworth, Wm. P.; May, Wm. B.; Woodruff, Timothy L. . . . . 379
Mathews, Joseph M; Lewis, Daniel; Johnston, Geo. Ben; Gihon, Albert L.; Krauss, William C.; Gibbons, Henry Jr. . . . . . . . . . . . . 64
McMurtry, L. S. . . . . 63
Miller, Jacob; Daggett, Byron H. . . . . . . . . . 380
Park, Roswell; O'Brien, E C. W.; Mathews, Joseph M.; Johnson, Ray H.; Lewis, F. Park . . . . . . . 871
Pryor, John H.; Frederick, C. C.; Dagenais, A.; Himmelsbach, G. A.; Bradley, H. H; Wile, Charles; Parmenter, John; Waterman, J. H.; Krauss, William C.; Brush, Edward N.; Werder, X. O. . 137
Ross, James F. W.; Bagley, Thomas . . . . . . 65
Walker, Francis A.; Holmes, J. B. S. . . . . . . . . . . . 138
Ward, Milo B.; Osthues, Henry. 940
Wise, P. M. . . . . . . . 316

Pertussis, Treatment of . . . . . 449
Physicians, Fraternal Relations of . . . . . . . . . . . . . 663
Physicians Should Work Less . . . 159
Pleural Effusion in Children 331
Pneumatic Cabinet, Forcible Breathing and the . . . . . . . 178

PAGE.

Pneumonia, Senile . . . . . . . . . 448
Prognosis in . . . . . 848
Polyclinic Hospital, Free Room in f r Physicians . . . . . . . 782
Poor-houses, Inspection of . . . . 612
Porro's Operation . . . . . . . . . 296
Potter, William Warren, American Public Health Association, A Historical Sketch, History of the Preparation for the Buffalo Meeting, 1896 . 105
Reciprocity in Medical Licensure; a Plea for Interstate Indorsement . . . . 883
Pregnancy Following Imperfect Coitus . . . . . . 347
Ruptured Tubal . . . 355
Prepuce, Dilatation of . . . . . 214
Putnam, Hon. James O., Remarks at Semicentennial Commemoration of Anesthesia . . . . . . 261

**Progress in Medical Science:**

Genito-urinary and Syphilitic Diseases, by B. H. Daggett. 38, 211, 348, 439, 595, 690
Medical Education and State Medical Examinations, by William Warren Potter. . . . 44, 442, 588, 857
Medicine, Pathology and Therapeutics . . . . 448, 848
Neurology, by Wm. C Krauss. 603
Obstetrics, Gynecology and Pelvic Surgery, by William Warren Potter . . . 352, 767
Ophthalmology, by A. A Hubbell . . . . . . 50, 358, 763
Pediatrics, by Maud Josephine Frye . . 119, 435, 600
Physiological Chemistry, by John A. Miller . . . . . . 33
Surgery, by John Parmenter. . . . . . . . . . . 121, 303, 684

Prostate, Castration for Hypertrophy of . . . . . . . . . . . . 39
Prostate, Inflammatory Disease of . 491
Prostatectomy for Prostatic Enlargement . . . . . . . . . . 598
Prostitution, Medical . . . 442
Protonuclein in Ulcer of the Stomach . . . . . . . . . . . . . 219
Pryor, J. H., Forcible Breathing and the Pneumatic Cabinet . . . 178
Pterygium, Etiology of . . . . . . 56
Pterygium, New Method of Treating . . . . . . . . . . . . . . . 364

PAGE.

**Public Health and Hygiene:**

Bubonic Plague at Bombay . 781
Consumption, Deaths from . . 781
Exercise, Exact Dosage in . . 146
Health Department, Victory for . . . . . . . . . 781
Public Health Conferences . 615
State Board of Health, Monthly Meeting . . . . . . . . . 145
Puerperal Insanity . . . . . . . . 352
Eclampsia . . . . . . . 353
Pupil, Formation of Artificial . . . 358
Putnam, Hon. James O., Remarks at Semicentennial Commemoration of Anesthesia . . . . . . . 261
Pylephlebitis, Suppurative of . . . 122

RACHITIS, Elimination of Calcium Compounds in . . . 35
Rafter, George W., On Lake Erie as a Water Supply . . . . . . . 10
Railway Accident, the First . . . . 159
Railway Surgeons are Exempt . . 444
Reciprocity in Medical Licensure . 883
Reed, Chas. A. L., Melano-sarcoma of the Female Urethra . . . . . 344
Remarks at Semicentennial Commemoration of Anesthesia . . 261
Retina, Photography of . . . . . 364
Retinitis, Albuminuric . . . . . . 365
Requirements, Preliminary vs. Final 594
Respiratory Metabolism . . . . . 35

**Reviews:**

Abbott: Principles of Bacteriology . . . . . . . . 76
Allingham: Diseases of the Rectum . . . . 320
Allis: Dislocations of the Hip 152
Allport: The Eye and its Care . 634
Annual Report of Michigan State Board of Health 156
Annual Report Illinois State Board of Health . . . . . . 397
Annual Report of Commissioner of Education, 1892-93, 72; 1893-94, 478; 1894-95, 630
Annual Report State Board of Charities, 1895 . . . . . 632
Annual Report of Pennsylvania State Board of Health 1894, 155; 1895 . . . . . . 633
Annual Report State Board of Health, '94, 148; '95 . . . . 879
Bartholow: Materia Medica and Therapeutics . . . . 555
Boisliniere: Obstetric Accidents, Emergencies, etc . . 152

PAGE.

Bosworth: Diseases of the Nose and Throat . . . . 554
Buck: Vest Pocket Medical Dictionary . . . . . . . . 477
Buret: Syphilis in Middle Ages and Modern Times . 75
Butler: Materia Medica, Therapeutics, etc . . . . . . . . 557
Canfield: Urinary Analysis . . 558
Chapman: Medical Jurisprudence and Toxicology . . 76
Corwin: Diagnosis of the Thorax . . . . . . . . . . 634
Culbreth: Materia Medica and Pharmacology . . . . . . 475
Curtis: Voice Building and Tone Placing . . . . . . . 73
Davis: Treatise on Obstetrics . 548
Deaver: Treatise on Appendicitis . . . . . . . . . . 472
Delafield-Prudden: Pathological Anatomy and Histology . 552
Dennis: System of Surgery, Vol. IV. . . . . . . . . . 147
Dictionary, Lippincott's . . 943
Dorland: Manual of Obstetrics . . . . . . . . 550
Edwards-Harraden: Two Health Seekers in California 796
Einhorn: Diseases of the Stomach . . . . . . . . . . . 718
Ewald: Diseases of the Stomach . . . . . . 881
Gage: Microscope and Microscopical Methods . . . . 793
Gant: Diseases of the Rectum, Anus, etc. . . . . . . . . . 150
Gould: Students' Medical Dictionary . . . . . . . . 393
Gould: Borderland Studies . 393
Gould-Pyle: Anomalies and Curiosities of Medicine 719
Gould: American Year-Book of Medicine and Surgery . . 629
Gray: Descriptive and Surgical Anatomy . . . . . . 394
Hare: Practical Diagnosis . . 470
Hatfield: Diseases of Children . . . . . . . . . . . . 320
Hatfield: Compend of Diseases of Children . . . . . 153
Hayes: How to Live Longer, etc. . . . . . . . . . . . . . 794
Haynes: Manual of Anatomy . 72
Hayden: Venereal Diseases . 477
Hyde: Syphilis and Venereal Diseases . . . . . . . . . . 390
Hyde: Diseases of the Skin . . 881
Hibbard: Diet for the Sick . . 321
Hogan: How to Feed Children. 74

PAGE.
Holt: Diseases of Infancy and Childhood . . . . . . . . 720
Howell: American Text-book of Physiology . . . . . . . 471
Huntington: Index, Catalogue, Surgeon-General's Office Library . . . . . . . . . . . 478
International Clinics: Vol. IV., Fifth series, 149; Vol. III, Sixth series, 556; Vol. I., Seventh series . . . . . . 944
International Medical Annual and Practitioner's Index . . 796
Jackson: Diseases of the Skin . 478
Keil's Medical Register-Directory . . . . . . . . . . . 155
Kirstein: Autoscopy of the Larynx and Trachea . . 792
Loomis-Thompson: System of Practical Medicine . . . 790
Lydston: Over the Hookah . . 791
McGillicuddy: Disorders of the Nervous System in Women . . . 476
Morris: Diseases of the Genital and Urinary Organs . . 70
Morris: Lectures on Appendicitis . . . . . . . . . . . 880
Morris-Oliver: Diseases of the Eye. . . . . . . . 627
Murrell: Pharmacology and Therapeutics . . . . . . . 555
Musser: Medical Diagnosis . 476
Park: Treatise on Surgery . . 385
Presbyterian Hospital Report . 75
Pye: Bandaging and Dressing. 795
Remsen: Theoretical Chemistry . . . . . . . . . . . 942
Rockwell: Uses of Electricity . 559
Roosevelt: In Sickness and in Health . . . . . . . . 234
Savage: New Truths in Ophthalmology . . . . . . . . 236
Schaeffer: Atlas of Obstetrics . 631
Simon: Clinical Diagnosis, etc . 553
Saundby: Renal and Urinary Diseases . . . . . . . . . 791
Stedman: Twentieth Century Practice, Vol. VII., 553; Vol. VIII, 318; Vol. IX., 878; Vol. X. . . . . . . . . . . 788
Stevens: Practice of Medicine. 795
Sternberg: Text-book of Bacteriology . . . . . . . . . 236
Stewart: Diseases of the Male Urethra . . . . . . . . . 715
Storrey: Points for Nurses in Private Practice . . . . . 237

PAGE.
Transactions: Medical Society of the State of North Carolina. . . . . . . . 73
American Surgical Association, Vol. XIII., 74; Vol. XIV. . . . . . . . . 793
American Microscopical Society. . . . . . . . . 75
American Laryngological Association, '95, 151; '96. 879
American Association of Obstetricians and Gynecologists, Vol. VIII. . . 151
American Academy of Railway Surgeons . . . 397
American Gynecological Society, Vol. XXI . . . 635
American Dermatological Association, 1895 . . . . 154
Colorado State Medical Society . . . . . . . 636
Medical Association of Central New York. . . 156
Medical and Chirurgical Faculty of Maryland 635
Medical Society of Virginia . . . . . . . . . 634
Medical Society State of Pennsylvania . . . . . 557
Medical Society State of New York, 1896. . . . . 153
Philadelphia County Medical Society. . . . . . . 155
Rhode Island Medical Society . . . . . . . . . 154
Southern Surgical and Gynecological Association, Vol. VIII. . . . . 235
Texas State Medical Association. . . . . 631
Treves: A System of Surgery, Vol. II. . . . . . . . . . . 468
Turnbull: Artificial Anesthesia 792
Tussey: High Altitude Treatment of Consumptives . . . 794
Tyson: Practice of Medicine . 624
Vaughan: Ptomains, Leucomains, etc . . . . . . . . . 621
Visiting Lists for 1897: The *Medical News*, the *Medical Record* and Lindsay & Blakiston's . . . . . . . . 395
Weekly Abstract of Sanitary Reports . . . . . . . . . . 154
Wells: Compend of Gynecology . . . . . . . . . . . . 153
Wharton: Minor Surgery and Bandaging . . . . . . . . 551
Wilder: Neural Terms . . . . 795

PAGE.
Wilson: Text-book of Applied Therapeutics . . . . . 396
Wise: Text-book for Nurses . 632
Witthaus-Becker: Medical Jurisprudence, Vol. IV. . . 547
Wood-Fitz: Practice of Medicine . . . . . . . . . . 473
Year-Book of Treatment for 1897 . . . . . . . . . . . 794
Yeo: Food in Health and Disease . . . . . . . . . . 474
Rhode Island, Itinerant Physicians not Allowed to Practise in . . . 445
Richmond, Charles H., Antiseptic Treatment of Typhoid Fever . . 586
Richmond, Nelson G, Acute Epiphysitis in Infants . . . . . . 161
Rider, Wheelock, Rational Treatment of Asthenopia . . . . . 580
Roe, John O., Breathing and Bicycling . . . . . . . . . . . . . 339
Röntgen Rays in Location of Foreign Bodies in the Eye . . . . . 359
Rulison, E. S., Should the Marriage Contract be Limited by Law? . . . . . . . . . . . . . 733

SAMO, James B., In Memory of . 677
Sanitary Triumph, Buffalo's . 62
Sanitation, Discussion of in Popular Assemblies . . . . . . . . . . 314
Satterlee, R. H., Coloboma of the Iris . . . . . . . . . . . . 280
Schools, Medical Inspection of . . 458
Sciatica, a Neuritis . . . . . 410
Abolition of Reflex in . . 605
Scopolamine in Plastic Iritis . . . 362
Sefton, Frederick, Medical Experts and Expert Testimony . . . . . 403
Snow, Cleaning of From Streets . 533
Irving M., Antitoxin Treatment of Diphtheria in Buffalo . . . . . . . . . . 723

### Society Meetings:

American Association of Obstetricians and Gynecologists . . . . . 65, 142, 383, 940
American Public Health Association . . . . . . . 68, 143, 786
American Dermatological Association . . . . . 69
American Academy of Railway Surgeons . . . . 138
American Electro-Therapeutic Association . . . . . 143
American Association for Advancement of Science . . . 144

PAGE.
American Microscopical Society . . . . . . . 231
American Gastro-Enterological Association . . . . . . . 875
American Medical Publishers Association . . . . . . 786, 942
American Medical Editors' Association . . . . . . . . 787
American Institute of Homeopathy . . . . . . . . . 786
American Academy of Medicine . . . . . . . . 787
American Medical Association. . . . . . . . . . . . . 714, 785
American Laryngological, Rhinological and Otological Society . . . . . . . . . 384
Association of American Medical Colleges . . . . . . . . 787
Association of Erie Railway Surgeons . . . . . . . . . 231
Association of Military Surgeons of the U. S. . . . 942
Buffalo Academy of Medicine. 229, 382, 466, 543, 619, 714, 785, 874 . . . . . . . . . 941
Buffalo Sanatory Club . . . . 715
Congress of American Physicians and Surgeons . . . . 787
French Association of Urologists . . . . . . . . . . . 875
Lake Erie Medical Society. . . . . 318, 542, 786
Medical Society State of Pennsylvania . . . . . . . . 787
Medical Society County of Cattaraugus . . . . . . 874
Medical Society State of Wisconsin . . . . . . . . . . 787
Medical Society County of Westchester . . . . 544
Medical Society State of New York . . . . . . . . . . 230, 464
Medical Association of Central New York . . . . 226, 230, 318
Medical Society of the County of New York . . . . . . 383
Medical Society of the County of Erie . . . . . . 467, 675, 875
Medical Society of the County of Chautauqua. . . . . 468, 542
Mississippi Valley Medical Association . . . . . . . . 142, 231
Missouri State Medical Association . . . . . . . . . 715, 786
National Confederation of State Medical Examining Boards . 784
New York State Association of Railway Surgeons . . . . . 231

PAGE.
New York Academy of Medicine . . . . . . . . . . . . 620
Ohio State Medical Society . . 941
Pan-American Medical Congress . . . . . . . . . 233, 383
Southern Surgical and Gynecological Association . . 317, 381
Tri-State Medical Society . . . . . . 143, 232, 543, 714
Twelfth International Medical Congress . . . . . . . . 466, 715
Western Ophthalmological, etc. Association . . . . . 468
Western Surgical and Gynecological Association . . . 384

**Society Proceedings:**

American Public Health Association . . . . . . . . . 198
American Association of Obstetricians and Gynecologists . . . . . . . . . . . 281, 743
Medical Society County of Erie . . . . . . . . . . 264, 526
Medical Society, County of Chautauqua . . . . . . . 32
Medical Association of Central New York . . . . . . . . . 515

Sodium Iodide, from Blood to Lymph . . . . . . . . 37
Salicylate and Uric Acid . 38
Spitting in Street Cars . . . . 534, 782
Sprained Ankles . . . . . . . . 121
Stanton, William, Clinical Report, by . . . . . . . . . . . . . . . 923
State Board of Pharmacy . . . 861
State Medical Societies, What They Have Done . . . . . . . . 933
Stephenson, F. H., Two Cases of Friedreich's Ataxia 568
Strabismus, Treatment of Convergent . . . . . . . . . . 50
Stockton, Charles G , Some Aspects of Enteroptosis . . . . . . . . 895
Sturtevant, J. R., Fraternal Relation of Physicians . . . . 663
Substitution by Unscrupulous Druggists . . . . . . . . 60
Succus Entericus of Sheep . . . . 34
Sun-stroke in 1896 . . . . . . 855
Surgery of the War . . . . . . . 563
Surgical Cases, Points in After-treatment of . . . . . . . . . 836
Syphilis, Serum Treatment of . 38, 440
Treatment of Advanced . 439
Syphilo-dermata, Treatment of . . 690

PAGE.
TACHYCARDIA, Paroxysmal . 848
Tait, Lawson, Cleansing and Cleanliness in Abdominal Operations . . . 483
Was Robert Houston the First Ovariotomist? . . . . . . 803
Thompson, J. G., Surgery of the War, 1861–1865 . . . . . . . . . 563
Thyroid Gland, Uses of . . . . . 437
Sexual Relation of. 769
Extract in Chorea . . . . 438
Topics of the Month . . . 60, 225, 313, 373, 456, 533, 612, 703 . . . 780
Toxic Substance from Suprarenal Capsules . . . . . . . . . . . 34
Trional, Unpleasant Effects of 849
Tuberculosis . . . . . . . . . . . 851
Treatment of Vomiting in . . . . . 701
Diagnosis of . . . 850
Tuberculous Disease, Abdominal Section for . . . . . . . . . 300
Tuboövarian Cysts . . . . . . . 286
Typhoid Fever from Ice Cream . . 449
Antiseptic Treatment of . . . . 586
Serum Diagnosis of. 847
Treatment of . . . 854
Ulcer, Surgical Treatment of Perforating . . . . . 687

ULCER of the Stomach Treated with Protonuclein . . . . . 219
Ullman, Julius, Clinical Importance of Examination of Feces . . . . 89
Urea in Animal Organs . . . . . . 37
Uremic Dyscrasia, Alteration of Nerve Elements in . . . . . 603
Urethra, Melano-sarcoma of Female . . . . . . . . . . . . 300
Urethral Electrolysis . . . . . 595
Urinary Sediment, Preservation of. 440
Urine, Quick Filtration of . . . . 349
Testing of . . . . . . . 439
Operation for Incontinence of . . . . . . 770
Uriniferous Tubules, Casts of . . 1
Uterine Fibroid Operation . . . . 923
Uterine Myoma, Case of . . . . . 511
Displacements, Etiology and Pathology of . . 667
Uterus, New Method of Dealing with Rupture of . . . . 61
Rupture of . . . . . . . 295
Cancer of Pregnant . . 354

PAGE.

VAGINAL Route vs. Abdominal Section in Periuterine Septic Diseases . . . . . . . . . . . 743
Varicocele, Radical Cure of . . 441
Vessels, Suture of . . . . . . . . . 301
Virginia, New Members of Medical Examining Board . . . . . . . 448
Vital Statistics, New Blanks for . . 375
Vitreus, Röntgen Rays in Foreign Bodies in . . . . . . . . . 755
Vivisection vs. Antivivisection . . 366
in the District of Columbia . . . . . . . 561
Voice-Users, Ailments of . . . . . 920
Vulvar Vegetation . . . . . . . . . 852

PAGE.

WATER Supply, Lake Erie as a . . . . . . . . . . 10
Wende, G. W., Mycosis Fungoides, with Report of a Case . . . . 832
Williams, H. T., Parasites in the Vermiform Appendix, with Case . . . . . 176
Laparatomy and Drainage in Purulent Peritonitis . . . . . . . . 509
Woman's Place in Medicine . . . 868

YOUNG, A. A., The Physician and His Microscope 195
Herpes Facialis . . . 651

